# Remarks on the Surgical Practice of Paris. Illustrated by Cases. Being a Thesis to Which a Gold Medal Was Assigned by the Senatus Academicus of the Edinburgh University at the Graduation of 1840

**Published:** 1841-01

**Authors:** 


Art. II.—*Remarks on the Surgical Practice of Paris. Illustrated by Cases. Being a Thesis to which a gold medal was assigned by the Senatus Academicus of the Edinburgh University at the Graduation of* 1840. By W. O. Markham, m.d.—*London*, 1840. 8vo, pp. 114.

This work appears to be little more than a series of scraps selected from the clinical lectures of various Parisian professors, and put together with little order or arrangement. Did we feel that the opinions of the author were worthy of serious consideration we should go through the work and criticise the treatment said to be practised in the hospitals of Paris. But as this is not the case, we merely select a few extracts, on points of some interest, which appear to contain rather statements of fact than matter of opinion. We commence with a method of treating abscess, said to be pursued by M. Roux, which appears most unwarrantably severe.

"M. Roux has a treatment of some kinds of abscesses which, I should imagine, is quite peculiar to himself. His method is to extirpate the whole of it or all of it that he can conveniently. Thus, where an abscess has been situated on the knee, in front of the patella, and caused by a fall, I have seen this gentleman make an incision on either side of the patella, in the axis of the limb, then join these two by a cross incision over the middle of the patella, and dissect up and down, and cut off the flaps above and below this cross incision; also in a scrofulous abscess of a gland in the axilla. I have seen him enlarge the free incisions already made in a crucial sense, dissect back the four angular flaps, and then cut them each severally off." (p. 10.)

According to our author, the French hospital surgeons occasionally commit gross blunders. We observed one or two instances in the work which will appear startling to English practitioners. For instance, Dr. Markham says he has seen M. Blandin "thrust the trocar into the testicle in a small hydrocele, and where, by the aid of a light, the testicle could be distinctly seen lying at the back of the scrotum, on withdrawing the trocar, no liquid escaped, and examination, with a light, showed the trocar sticking in the testicle." (p. 19.) M. Ricord observed that once, in a great hurry, he sent the trocar right through the testicle of a barber. (*Ib.*) M. Ricord once injected a quantity of wine into the peritoneum without a shade of a bad result, and he mentioned that he remembered M. Richerand having done the same thing. (p. 22.) M. Ricord said that he had seen Dupuytren and his own predecessor at the Hôpital du Midi, cut off the glans penis as well as the prepuce, in operating for phymosis by circumcision." (p. 76.) Some dreadful accounts are also given of the results of M. Louvrier's operations for anchylosis, but Dr. Markham says he has seen several cases of anchylosis of the knee admirably treated by M. Lisfranc, by means of a gradually extending apparatus. We are told that "the treatment of nearly all kinds of fractures by the starch bandage is now almost generally adopted by the Parisian surgeons," and that "according to my judgment it is one of the greatest acquisitions of modern surgery." (p. 70.) Autoplastic operations appear to be boldly practised. The following case is related:

"M. Blandin operated on a man who, in his youth, had had a malignant pustule beneath the left eye, which had destroyed the whole of the lower eyelid and a portion of the face below it; of course the eye always remained in great part uncovered, and when he came to the Hôtel Dieu, there was, as might be expected, much inflammation in the conjunctiva, and two or three small ulcers on the cornea. M. Blandin dissected a portion of healthy skin from the temporal region, commencing just below the outer angle of the eye, and continued the dissection upwards as high as necessary; he then turned round the portion and united it to the surface freshly denuded below the eye. The operation succeeded perfectly; it enabled the patient to close his eye, and removed all the deformity which existed before." (pp. 79-80.)

The only other remarks we think worthy of extraction are on a subject now beginning to excite considerable attention, that of animate contagion.

"The presence of animalcules in the discharge of chancres has been determined by Dr. Donné, and by him supposed to be the veritable specific virus—a species of vibrio (vibrio lineola of Müller), has been observed by him in ulcers of the gland, prepuce, vulva, and vagina (and the same also in the pustules of inoculation;) myriads appear to exist in a little drop. Another and peculiar species has been determined in the secretion of the vagina. Dr. Donné is led to suppose that the cause of chancres and gonorrhœa are these vibriones, and hence that their cause is identical; but no vibrio has yet been seen in the discharge of gonorrhœa." (p. 97.)

In concluding our notice of this book, we are constrained to state that the fact of the gold medal of the University of Edinburgh being assigned to it says very little for the merit of rival essayists. The author in his own preface gives a very correct character of his work, stating that it is "at best but a partial view of the matter, written desultorily, without any distinct purport." Under these circumstances it may be questioned whether its publication is not something more than injudicious.

